# Anæsthetics

**Published:** 1885-09

**Authors:** Frank W. Ryan


### THE DENTAL REGISTER.

VOL. XXXIX.] SEPTEMBER, 1885. [No. 9.

### COMMUNICATIONS.

### Anæsthetics.

BY FRANK W. RYAN, D.D.S.

We believe that the all-wise and merciful Deity, who created us with such a high state of sensibility, has also provided us with the means of allaying those degrees of sensibility which we designate as pain. And we think it to be the duty of every man to contribute as much as he can toward the mitigation of those ills which are the result of our own and our parents' disobedience to the laws of health. But we more particularly believe it to be the duty of those gentlemen who have made a study of the human organism, both in health and disease, and to whom humanity at large apply for relief when the system is in an abnormal condition.

We, as dental surgeons, should bestir ourselves to become well acquainted with the various agents which have been found to possess the property of obtunding pain, because of the fact that, besides our common duty to humanity, in very many of the operations we are called upon to perform, in order to relieve our fellow men, we cause the most excruciating pain.

We have quite a number of agents that have been found to cause loss of sensation, some to be applied locally, others administered to the system generally, but to all of which serious objections may be taken. The man who discovers an agent, or means, which will cause loss of sensation without loss of consciousness, and which will embrace a number of the many good qualities of the present anæsthetics, will achieve a degree of fame that will not die with him, but which will run down the pages of medical literature for many generations to come.

According to the best authorities, it would seem foolish, at this late date, to suffocate ourselves or patients with "aldehide," suffer the headache consequent upon the use of "nitrate of ethyl," subject anyone to the miserable stench of "bisulphuret of carbon," or be thrown into convulsions by the use of the vapor of "benzoin."

These have all been found unprofitable servants, and decidedly bad as masters.

Among the more important anæsthetics of the present day, we may mention sulphuric ether, chloroform, and nitrous-oxide gas.

Sulphuric ether, which probably stands at the head of anæsthetic agents in this country to-day, has a chemical formula of $C_4H_5O$. It was first used as an anæsthetic by Dr. Horace Wells, a dentist, of Hartford, Conn., in the year 1845.

It is a transparent, colorless liquid, with a strong, fragrant odor, and a hot, pungent taste. It wholly evaporates in air, and is very inflammable. It combines with alcohol and chloroform in every proportion, and dissolves in ten times its volume of water. Boils at 96° F. Its specific gravity is .713. Although this substance is among the lightest of the fluids its vapor is very heavy, having a density of 2.586.

When the vapor of this substance is inhaled and absorbed through the pulmonary surfaces, the nervous functions are successively and progressively affected. First, there is commonly exhilaration, this shading gradually into stupor. The mental faculties and volition first become impaired, insensibility and unconsciousness rapidly supervene, during which susceptibility to pain is lost, and the patient lies in a trance-like sleep resembling death.

As the stage of insensibility is reached, convulsive twitches are sometimes noticed, the breathing becomes stertorous, the iris becomes fixed, pupils dilated, the eyeballs turned up, and the orbicularis palpebrarum muscle does not contract when touched.

It should not be administered when disease of the heart, or brain, or serious obstruction of the lungs exist, or when, from any cause, there is unusual tendency to syncope; and precaution should be taken to guard against asphyxia.

The point of danger relates with the respiration.

Easy breathing is assurance of safety.

The medulla oblongata is the last of the nervous centers to become affected, but when affected, it exerts a very serious trouble, and we may see our patient die beneath our hands from lack of respiration.

But, when administered with proper discrimination, and care to admit atmospheric air into the lungs along with it, there is little or no danger. Patients usually recover without serious inconvenience, although headache, nausea, drowsiness and langor sometimes ensue for a few hours. Occasionally congestion of the brain or lungs, cataleptic rigidity, with profound insensibility, and, in females hysterical phenomena ensue after etherization. But these effects are uncommon, and it is believed that death has never been caused by the use of ether, when care was taken in its administration.

"Chloroform" with a chemical formula of $C_2 H Cl_3$ was discovered simultaneously in New York and Paris, in the year 1847. It is usually obtained from the distillation of alcohol with chlorinated lime; but for medicinal use, the chloroform of commerce requires purification, which is accomplished by shaking it with sulphuric acid. The purest chloroform is now made from "Hydrate of Chloral." It is a colorless, volatile liquid, of a bland, etherial odor and a hot, aromatic, saccharine taste. It is not inflammable, is slightly soluble in water, and freely so in alcohol and ether. Has a specific gravity of 1.50 and boils at about 144° F.

The effects of the inhalation of the vapors of chloroform are analagous to those of ether, but much more rapid and powerful. When the vapor of chloroform is inhaled in the dose of a fluid drachm or more, it rapidly induces anæsthetic sleep, with great relaxation of the muscles, and the most complete insensibility to painful agents.

The period at which insensibility occurs, varies from fifteen seconds to two minutes, and may be prolonged considerably by renewals of the inhalation. The patient usually recovers without recollection of what has occurred during the state of insensibility, and with few or no unpleasant sequilæ.

The administration of chloroform has in some cases been attended with fatal syncope. This has ordinarily occurred with such rapidity, as to render remedial interference unavailing, but at the slightest approach of symptoms of the kind, the patient should be placed in a recumbent position, cold effusions should be applied, and above all electro-magnetism should be resorted to.

Chloroform is a specific narcotic so for as the heart is concerned, at least this is the deduction from post mortems made in the fatal cases of its exhibition. It relaxes and deadens the muscular fibres of the heart, causing death by the stoppage of the circulation.

"Nitrous oxide gas," chemical symbol $N_2O$, was discovered by Priestly, in 1776. Credit for its use as a pain obtunding agent is due to Dr. Wells, of Connecticut.

It is made from pure nitrate of ammonium by applying heat until about 250° F. is reached, when the salt fuses. The salt melted, the heat is increased to 460° F. the boiling point. If the gas is disengaged and passes off with freeness, the heat is to be maintained, if not it may be increased to 482° F., the maximum heat. When heated higher than this, poisonous elements are given off. It is purified before using by washing through various liquids. It is colorless and transparent, has a faint, sweetish smell and taste. It is unfit to support life, but may be breathed for a short time, when it exerts a remarkable influence on the nerves. If breathed in a pure state, it produces transient insensibility, and surgical operations may be performed without pain.

When breathed it produces anæsthesia by depriving the blood of oxygen, and causes sensations something similar to those experienced in drowning. Insensibility is quickly induced and as quickly passes away. Judgment of the effect of the gas is derived from observing the mucous surfaces.

Spasm of the glottis and syncope are the commonest interruptions. In both cases immediate attention is required to the tongue ; the organ to be seized with a dry napkin and drawn forward. In spasms, the placing of the tongue, allowing a few inhalations of air, is sufficient for relief. In syncope, the patient is placed in a reclining position ; fresh air is freely admitted ; water is dashed against the face ; smelling salts or ammonia fortior is applied to the nose, and various other means of resuscitating are employed. The danger lies in venous congestion. The patient becomes black in the face, which indicates danger, when the inhaler must be taken away, and the administration interrupted.

Within the last year, the local anæsthetizing properties of "Cocaine" have been more thoroughly studied, and have occasioned no inconsiderable stir in the medical world. It has been experimented with by various dentists, as to its value to the dental profession with varying results, some lauding its local anæsthetizing power while others condemn it as utterly useless in almost all cases. We hope there will yet be found a way of using this agent, especially by dentists, so that we may feel that a want long felt has at last been supplied.

